# Nurses’ experiences in caring for people with mental health problems hospitalized due to clinical comorbidities

**DOI:** 10.1590/0034-7167-2023-0136

**Published:** 2024-12-13

**Authors:** Gabriella Paulão Previato Piton, Aldair Weber, Ana Paula Rigon Francischetti Garcia, Vanessa Pellegrino Toledo

**Affiliations:** I Universidade Estadual de Campinas. Campinas, São Paulo, Brazil

**Keywords:** Comorbidity, Comprehensive Health Care, Psychiatric Nursing, Nurses, Mental Disorders, Comorbilidad, Atención Integral de Salud, Enfermería Psiquiátrica, Enfermeros, Trastornos Mentales

## Abstract

**Objectives:**

to understand nurses’ experiences in caring for people with mental health problems hospitalized due to clinical comorbidities in non-psychiatric Inpatient Units.

**Methods:**

qualitative study, guided by Alfred Schutz’s social phenomenology. Sixteen phenomenological interviews were conducted. The content was analyzed and discussed based on the literature, through the composition of three categories of analysis.

**Results:**

three categories emerged in the study: Challenges in care faced by nurses; Fragmented care action; and Ideal care. The disarticulation of the clinic was revealed, as described by nurses, showing care as an action far removed from the comprehensiveness of a person. Nurses’ performance is guided predominantly by biomedical reference, disregarding appreciation of subjectivity.

**Final Considerations:**

it was observed that nurses attribute the responsibility for patient care to factors external to their life-world, when, in fact, these aspects should be components that help them in comprehensive care construction.

## RESUMEN

**Objetivos:**

comprender las experiencias de enfermeros en el cuidado de personas con sufrimiento psicológico internadas por comorbilidades clínicas en Unidades de Internación no psiquiátricas.

**Métodos:**

estudio cualitativo, guiado por la fenomenología social de Alfred Schutz. Se realizaron 16 entrevistas fenomenológicas. El contenido fue analizado y discutido con base en la literatura, a través de la composición de tres categorías de análisis.

**Resultados:**

emergieron tres categorías en el estudio: Desafíos de cuidado enfrentados por los enfermeros; La acción fragmentada del cuidado; y Atención óptima. Se reveló la desarticulación de la clínica, descrita por los enfermeros, destacando el cuidado como una acción alejada de la plenitud de la persona. Las acciones de los enfermeros son guiadas predominantemente por el marco biomédico, desconsiderando la apreciación de la subjetividad.

**Consideraciones Finales:**

se observó que los enfermeros atribuyen la responsabilidad por el cuidado del paciente a factores externos a su mundo de vida, cuando, en realidad, esos aspectos deben ser componentes que les ayuden a construir un cuidado integral.

## INTRODUCTION

The psychosocial care model of the Brazilian Health System (SUS - *Sistema Único de Saúde*), established and implemented in the context of the Psychiatric Reform, is marked by the expansion of the concept of the health-disease process, which considers the principles of comprehensiveness and multidisciplinary care^(1)^. It aims to seek a more comprehensive understanding of human beings so that the object of intervention shifts from the disease to a person in psychological distress^(1)^. In this context, to ensure comprehensive care, nurses are invited to look at the challenges arising from the health demands presented by the population with mental disorders, experiencing care situations for which knowledge has proven to be insufficient since training^(2)^.

Given the complexity of caring for people with mental health problems, it is observed that this population presents multiple challenges in care, exacerbated by increased risk of developing physical comorbidities resulting from reduced access to adequate health care, reducing their life expectancy^(3)^. It is worth noting that people with mental health problems have a higher risk of premature mortality due to the presence of cardiovascular diseases, and that living with a mental disorder increases the chances of developing metabolic syndrome, characterized by changes in the cardiovascular system, which may include dyslipidemia, abdominal obesity, hypertension and hyperglycemia^(4)^. This increased risk is present in several psychiatric conditions, including major depressive, bipolar affective, schizophrenia, anxiety, attention deficit/hyperactivity disorder and post-traumatic stress disorders^(4)^.

Moreover, risk factors for cardiovascular disease, such as smoking, lack of physical activity and an unhealthy diet, are physical conditions that account for approximately 70% of deaths in patients with schizophrenia or bipolar disorder^(5)^. Thus, with pharmacological interventions, a significant improvement in the psychological condition is observed, but the use of antipsychotic medications increases the risk of weight gain, dyslipidemia and diabetes mellitus, and these factors also intensify the possibility of developing some clinical comorbidity^(5)^.

The prevalence of clinical comorbidities in people with mental health problems has been increasing in health services, culminating in hospitalizations. This scenario may have repercussions on the care provided, as these patients have low adherence to the proposed treatment. Other factors also stand out, such as restricted access to health services. Mental health problems may limit professionals’ action in building comprehensive care practices, in which the principles of inclusion and equity are not always guaranteed for this group of patients^(6)^.

Therefore, in the challenge of dealing with the complexity of caring for people with mental health problems and their associated clinical comorbidities, it is important to know the experience of healthcare teams regarding the care provided. Studies conducted with Brazilian and Australian nursing professionals show that caring for hospitalized psychiatric and non-psychiatric patients is still a challenge to be overcome^(7)^. This challenge can be identified by factors such as routine of technical procedures, generating distance from patients due to the negative stereotype of psychiatry, and neglect of physical examination, in the case of a person in psychological distress, and mental status examination is not carried out in clinical units, which may indicate the fragmentation of both physical and mental care^(7)^.

This scenario highlights impacts in practice and, since training, continuing education is recommended through training in the different sectors of health services, from emergency, through psychiatric unit to intensive care^(7-9)^.

It is important to highlight the factors that constitute challenges in health care for this population, such as fragmentation of care, which involves the complexity of a person in psychological distress, the epidemiological scenario, the implications experienced by nursing teams when providing care, and the existence of distinct and competing care models, one based on biological care and the other based on comprehensiveness guideline. In this regard, to advance in the search for comprehensive care, it is important to consider the reproduction of the logic of established practice, which is marked by the biological perspective in the assessment process, causing a fragmented view of a person in psychological distress affected by a clinical comorbidity^(1,7)^.

This study is justified by the lack of discussion in the literature on care for people with mental health problems hospitalized due to clinical comorbidities. Many studies are developed with the aim of obtaining epidemiological data that address their greater risk of death compared to that of the general population^(3-6)^. It is extremely important to carry out studies that aim to understand the experiences of the actors involved in this care scenario, such as nursing professionals. Given the low adherence and difficult access, there are impacts on practice marked by the biological perspective, such as the routine of technical procedures and patient distancing, caused by the stereotype of psychiatry^(1,6-9)^.

## OBJECTIVES

To understand nurses’ experiences in caring for people with mental health problems hospitalized due to clinical comorbidities in non-psychiatric Inpatient Units (IUs).

## METHODS

### Ethical aspects

The study complies with the ethical aspects of research involving human beings, according to Resolution 466/12 of the Brazilian National Health Council, and was approved by the *Universidade Estadual de Campinas* Research Ethics Committee in 2018. Participants signed the Informed Consent Form (ICF) and the Voice Recording Consent Form. To ensure anonymity, participants were identified by the letter “N”, for nurse, followed by the number corresponding to the order of the interviews.

### Theoretical-methodological framework

From Alfred Schutz’s perspective, understanding the meaning of action occurs based on existential motives and actions of subjects in the life-world^(10,11)^. When investigating them, a double motivational character is found based on a set of “reasons for” and “reasons why”^(10,11)^. The “reasons why” are related to past experiences, contextualized from a subject’s stock of knowledge^(10,11)^. The “reasons for” are related to the objectives that one wishes to achieve and the intentionality of actions projected in the future^(10,11)^. Schutz seeks to understand the world in its intersubjective meaning through an analysis of in-person social relationships, taking into account that actions occur consciously and have a meaning for a person^(10-12)^.

### Study design

This is a qualitative study, developed based on Alfred Schutz’s social phenomenology theoretical-methodological framework^(11)^. The aim is to understand the experience lived by nurses in caring for people with mental health problems hospitalized for treating clinical comorbidities and the meaning they attribute to this action, taking the IU as their life-world, endowed with knowledge, subjectivity, singularity, determined biography and motivations^(10-12)^. Seeking to maintain the scientific rigor of qualitative research, the COnsolidated criteria for REporting Qualitative research (COREQ) recommendations were followed^(13)^.

### Study setting

The research was conducted in different IUs of a public university hospital located in the countryside of the state of São Paulo, which was the setting for the training, research and extension of researchers involved in this study. Its outpatient and inpatient units are staffed by diverse professionals from the multidisciplinary team who ensure patient care, seek new forms of care based on daily life and expand the institution’s involvement with civil society. This hospital is a tertiary reference for multiple specialties, providing health care to patients with different needs, who are followed up in the public health network of the metropolitan region of Campinas.

### Study participants

The participants were 16 nurses working in the IUs who had experienced caring for people with mental illness hospitalized for clinical comorbidity treatment. Inclusion criteria were being a nurse, working in adult IUs, and caring for people with mental illness hospitalized in IUs for the clinical comorbidity treatment. Exclusion criteria were being a nurse who was on vacation or on sick leave at the time of data collection.

### Data collection and organization

To approach participants, network sampling was used, known as snowball sampling, in which one participant indicates another^(14)^, with the first being selected by the IU supervisors. Eight supervisors were referenced as being the seeds^(14)^, and they indicated nurses who they considered to have already had contact with the study topic. The IUs that were the setting for data collection are orthopedics, neuroclinic, gastroclinic, general adult ward, emergency and specialties, and infectious diseases. Phenomenological interviews were used to approach the interviewees and, based on intersubjectivity, it was possible for the subject who experiences the phenomenon to express the meaning of their action^(10,12,15)^. Semi-structured, individual, non-directive interviews were conducted, allowing for including new questions that aim to explore the experiences in their entirety. They were recorded, with an average duration of 40 minutes and guided by the guiding questions: have you ever cared for people with mental distress who were hospitalized for clinical comorbidity treatment in the adult IU? Tell me about this care. What was your intention with the care provided? The data were collected by the main researcher, who was trained by the advisor about the phenomenological interview with the research group. The researcher did not maintain a direct relationship with the nurses in the units where the data were collected. This allowed for a distance between participants and the setting for data analysis, which was restricted to the time of data collection. Data collection took place at nurses’ workplace, on a pre-scheduled date and time, from June to September 2018, and ended when the researcher understood that his concern had been answered and the phenomenon had been revealed, and no new elements emerged from the interviews to constitute the categories that represent the typification of the experience lived by nurses in caring for people with mental distress hospitalized with clinical comorbidity^(16,17)^. It should be noted that all interviews were used for data analysis and discussion, with no sample loss.

### Data analysis

To analyze the statements, the methodological framework proposed by researchers in social phenomenology was used^(10,12)^. The data were analyzed and categorized by the main researcher and the other authors who contributed to the study without using software. After the interviews were transcribed, they were read in full in order to grasp the common meaning of nurses’ experience and characterize the typification of what was experienced^(10,12)^. Subsequently, a careful rereading of the interviews was carried out, aiming to create units of meaning^(10,12)^. The speeches were then organized into thematic categories, and the meaning of the action expressed by the “reasons why” was identified, evidenced by the “Challenges in care faced by nurses” and “Fragmented care action” categories. The “reasons for” were described in the “Ideal care” category. A comprehensive analysis and discussion of results were carried out, using assumptions from social phenomenology^(10,12)^ and scientific literature related to the study topic.

## RESULTS

### Challenges in care faced by nurses

In the motivation of the experience, it was evident that participants reported difficulties in caring for a person with mental distress hospitalized. This is a specialty that is not common in the IU, in addition to the fact that they do not have the same understanding that other patients have about caring for and recovering during hospitalization, which is characterized as a difference in nurses’ vision.

*We have difficulties, because it is a specialty that we do not have every day, right? Psychiatric patients stay there in their ward and, occasionally, they stay here.* (N1)

*He does not understand that he has to be restricted because of the fracture he has* [...] *if it is a lower limb fracture, it is complicated, because you will have to restrain patients, otherwise they will get up and fall. That is the difference with these patients we have.* (N8)

Nurses’ discourses also demonstrated some difficulties in the process of caring for patients in the IU. The absence of family members during hospitalization hinders a relationship of trust for care and discharge planning. The inadequate physical structure was also considered a difficulty in the process of caring for mental health in the IU, characterized by lack of protection, leisure and activities outside the bed.

[…] *these patients are usually left without family members, it is harder to build trust, and you cannot provide care without patients trusting you.* (N2)

*Family members are not always present, especially with substance abusers. In these cases, it is more difficult to plan discharge.* (N14)

*The physical structure is not suitable for treating not only psychiatric patients* […] *we are on the sixth floor, without much protection. In addition, we have no leisure facilities, no outdoor areas, no landscape or any activities outside the bed, nothing that actually provides mental health care.* (N10)

In this scenario, the interviewees discuss the risk of decompensation of patients’ psychiatric condition, which is compounded by the nursing team’s fear of providing care due to the risk of aggression. Therefore, when faced with the need to perform physical restraint, professionals are faced with a lack of supplies to carry it out, in addition to the absence of a doctor or a specific medical prescription, aspects that nurses consider need to be present in patient admission protocols in the IU.

[…] *if physical restraint is not prescribed, it makes our work much more difficult, because, at the time of the incident, doctors are not* […] *there is always this risk of decompensating the psychiatric part, there would have to be a protocol for the hospitalization of this type of patient.* (N7)

[…] *the nursing team is afraid to care for psychiatric patients, especially in those cases where patients become threatening.* (N8)

[…] *there is a lack of several things to care for psychiatric patients, material, bandages, training for physical restraint.* (N5)

Experiences such as psychomotor agitation and aggressiveness were classified as negative aspects of the experience of caring for patients with psychological distress in the IU, due to the risk of contamination, in addition to the lack of a team to respond to psychological care demands.

[…] *there are patients who attack employees, pushing them, trying to hold them back. Right now, there is a patient who had his access removed and splattered blood. He is HIV positive* [Human Immunodeficiency Virus], *he splattered blood on the ceiling, he splattered it on his colleague, it was really bad.* (N4)

[…] *there are aggressive and agitated patients and, sometimes, we don’t have a team that can provide this care, which involves the psychological part.* (N3)

Finally, interviewees point out that the lack of knowledge about psychiatric pathologies and the lack of preparation of nurses to care for hospitalized people with mental health problems raises questions about care management, approach and conduct.

*We don’t have adequate knowledge of pathologies. We don’t know how to manage them. How do I approach patients? How do I care for them? How far can I go*? (N12)

*It’s harder to care for these patients here. I think the team wasn’t prepared to care for psychiatric patients, I myself didn’t go to college.* (N15)

### Fragmented care action

It was evident that the emotional aspects of a person in psychological distress hospitalized in the IU are in a secondary care plan, and that, during nursing history data collection, nurses indicate that they only investigate the physiological aspects, directing the anamnesis towards hospitalization diagnosis.

[…] *in the nursing history, there are some issues in the emotional approach. As a cardiology nurse, I don’t give importance to this, I stay with the physiological. (*N16)

[...] *patients were hospitalized due to a DVT* [Deep Vein Thrombosis], *so if they are psychiatric, this ends up being secondary, and patients are known by hospitalization diagnosis.* (N7)

Fragmentation of individuals based on medical specialties removes the condition of nursing care regarding psychiatric issues during hospitalization in an ICU. Physicians are still responsible for requesting assessment and conduct through interconsultation.

*I think that orthopedic nurses are not qualified to care for psychiatric patients.* […] *but those who have more experience with these fracture complications are orthopedic nurses.* (N10)

[…] *I don’t know how psychiatric assessment really works. I know that interconsultations exist, and it is up to physicians to request them.* (N9)

During the hospitalization of people with mental health problems, nurses find it difficult to prescribe care that meets the demands related to mental health, wondering how to meet the need for emotional support. Therefore, when planning care, nurses use a roster among all team members so that no one feels wronged, in order to avoid fatigue and emotional exhaustion.

[…] *how will I prescribe for the team to care for this patient? “Give emotional support” is very open-ended. What does nursing mean by providing emotional support? If we, as nurses, have this difficulty in assessing, imagine technicians* […] *we don’t know exactly what to prescribe for this care, it gets confusing.* (N8)

[…] *it’s hard to plan who will provide care. We end up doing a roster schedule, so everyone needs to provide care and it’s not unfair to anyone.* (N6)

[…] *we aim to have a greater roster of nursing technicians, so that they don’t feel tired on the daily schedule. We think about this so that professionals don’t have emotional exhaustion.* (N9)

However, in the routine to be followed in the unit, administrative demands and the lack of human resources hinder the planning of nursing care. Consequently, nurses are further away from the care, so that nursing technicians are the professionals who are closest.

[...] *it is difficult because it already comes with a large demand. The routine gets in the way*. (N3)

[...] *lately, our bureaucratic work, in the administrative area, has led us to take on several service demands for which we no longer have employees. So, those who are closest to the patient end up providing more support, in this case, nursing technicians, and then they pass on to us information about the care they provided.* (N7)

As for care implementation, interviewees described compliance with medical prescriptions at pre-established times as the main factor in clinical treatment. However, the possibility of the team approaching patients to provide mental health care during prolonged hospitalization was mentioned.

[...] *to comply with medical prescriptions, clinical treatment, I believe that the administration of medication correctly, the correct prescription, has more weight* [...] *for the clinic, following all these pre-established care and schedules.* (N1)

[...] *if patients stay longer, you can get the team to talk, but sometimes this does not happen, they go home without care* [...] *in this way, we take care of the physical and clinical part, but the psychological part was not taken care of at all.* (N7)

### Ideal care

Nurses demonstrated a desire to have more time to talk, listen to patients and feel their difficulties as expectations for caring for people with mental health problems. Added to this is the desire of professionals to have more preparation and training and the need to share experiences to provide ideal care.

*I wish I had more time to talk, to do a job that we know one day is not enough, to arrive and listen every day you have to feel the difficulties.* (N1)

[...] *it would be important to be more prepared, to have more information about psychiatric illnesses. Courses, more information, so we are better prepared.* (N13)

*We could form a circle, bring a clinical case and talk about this mental part* [...] *talk about what is difficult, what makes care easier, because there are people who have positive things to say, which can help colleagues.* (N3)

[...] *I would like nurses to be trained in relation to this issue of psychiatric patients, trained in how to manage, how to deal with.* (N11)

In view of this, the interviewees understand that, at the time of patient admission, a multidisciplinary assessment is necessary so that their health needs are considered and also so that the nursing team knows more about how to care for them.

Moreover, the need for psychological support for the nursing team is identified as a way of caring for those who care, supported by the peculiarity of the profession in dealing with death and tragedies, factors that can lead to illness.

*I think the nursing team, as a whole, should have psychological support* […] *we deal with death, with many situations like that, tragedies. Some of the team has been in the hospital for over 20 years, so they get sick.* (N4)

*From the moment of admission, there should be a multidisciplinary team, a psychiatrist, psychologist, occupational therapist, and they should come to the ward to assess patients’ needs* […] *this assessment would be important for the team to know more about how to care for them.* (N8)

## DISCUSSION

In relation to the challenges faced by nurses when experiencing care for people with mental health problems hospitalized due to clinical comorbidities, professionals’ and patients’ life-world has a strong influence on nursing care, as the subjectivity of both must be considered^(10,11)^. Life-world, constituted from biographical aspects and the stock of knowledge, is present in the nurse-patient relationship, in which the action of care occurs as a result of such an encounter, permeated by mutual objectives and previously established between the main actors^(10,11)^.

The findings of this study indicate that the care of signs and symptoms resulting from clinical comorbidities is made difficult by the existence of a mental disorder, when there is a need for clinical hospitalization, as it brings specificities that are not commonly found in IUs. One difficulty can be listed as the lack of understanding about the need for hospitalization, creating a barrier to providing clinical care. This scenario is the result of fragmentation of a person, directly impacting care strategies and their expected results, since a mental disorder is seen as something separate and not as a component of the same individual’s biological organism^(7)^.

Furthermore, such fragmentation reproduces lived stigmas and fears that are heightened, such as aggression, linked to the lack of knowledge of how to care for a hospitalized person with mental distress, making the nurse-patient relationship difficult^(9,18)^. From this perspective, indirectly, nurses understand the importance of bonding and bring the family’s presence during hospitalization as an important strategy to assist in the different ways of caring for psychiatric patients hospitalized due to physiological changes^(19)^.

Another aspect highlighted by nurses, related to the challenges in caring for people with mental health problems hospitalized due to a clinical comorbidity, was the inadequate work environment, which, according to them, also impacts the lack of protection, leisure, activities outside the bed, and institutional support. Thus, it is important to consider that the IU environment constitutes part of the life-world of nurses participating in this study, influencing social relationships and their attitudes that guide how to care for patients. Hence, the implementation of strategies that improve the environment can aim at humanization of care in hospital settings^(20)^.

According to the perception of nurses involved in the study, it is considered important to prescribe mechanical and chemical restraint in cases where the assessment of a person in hospital distress from mental illness indicates the need for such a procedure. This approach may reflect a vision of health that responds to the biomedical model, focused on psychiatric diagnosis, leaving aside the assessment of a clinical condition that would justify such a prescription and the uniqueness of human beings^(21)^.

The rush to decide on the need for containment protocols, linked to the existence of a psychiatric diagnosis, is highlighted as an aspect that negatively impacts care, as seen by professionals’ experience of psychomotor agitation and aggressiveness displayed by hospitalized individuals suffering from mental illness. Violence and agitation, when considered in life-world, constituted by the stock of knowledge and biographical aspects of nurses, can justify the distancing of the subjectivity and singularity of each person, in which nursing care is legitimized by scientific knowledge, imposing a state of ideal and healthy normality regarding how people should be and behave^(10,11,22)^.

Based on Schutz, it can be said that there was no acquisition of knowledge related to care for hospitalized people with mental illness as part of the life-world of the nurses studied in their construction of stock of knowledge, characterized by the lack of technical-scientific preparation during the training period as well as from the perspective of continuing education. This gap in training is implicit in the difficulty for nurses to perform an integrated assessment of health status of people with mental illness hospitalized due to a clinical comorbidity^(7,10,11)^.

In this regard, Brazilian studies have highlighted the difficulties in caring for hospitalized people with mental health problems, since, even in specialized services for this population, a deficit was identified in relation to specific technical-scientific knowledge to train future professionals for mental health management. Barriers related to work processes were also observed, such as team exhaustion, the scrapping of services and the distance between professionals and patients^(21,23)^.

It was also possible to observe that nurses reproduced the reductionist and fragmented view of pathology based on a biological process, separating body and mind when admitting patients to the IU: they based themselves only on the stages of physical examination, excluding mental status examination, distancing themselves from patients’ assessment of aspects that go beyond the body. This finding supports a Brazilian study that highlighted the distancing of nurses in relation to hospitalized psychiatric patients, failing to perform physical examination and systematize the mental health care of clinical patients through mental status examination^(7)^.

Furthermore, when learning about nurses’ perceptions of clinical patients’ psychiatric symptoms, they report low training hours, identifying the need to qualify care through training, knowing psychiatric hospitalization units^(24)^. Hence, it is evident that attention to the psychiatric symptoms of hospitalized people with mental distress occurs on a secondary level to clinical care, characterizing fragmentation of care, identified by the absence of aspects related to nurses’ stock of knowledge about mental health and psychiatric nursing and their professional practice, with common sense and everyday concepts of the social world predominating^(7,10,11,24)^.

The experience described by nurses signals the life-world of nursing with the care for a hospitalized person in psychological distress. This world is shared among team professionals so that nurses remain in their already existing and constituted biographical situation, even if the need to motivate oneself in search of elements for the stock of knowledge is identified^(10,11)^. The emotions and feelings brought by interviewees in their reports about the experience with this care, such as fear of being attacked and discomfort in relation to psychiatric patients, can configure the in-person relationship, characterized as the encounter with another subject’s life-world, orienting each other with a greater possibility of mutual understanding^(9-11)^. For nursing care for hospitalized people with mental illness, Schutz’s in-person relationship is shown to be a possibility of alleviating the discomfort experienced by nurses in the nurse-patient relationship. It allows us to understand what sensations permeate this relationship from contact with life-world and its various constituent aspects up to the moment of this encounter^(10,11)^.

In this paradigm, the discourses of nurses participating in the study also highlighted the difficulty of planning and prescribing nursing care that includes patients’ mental health, regardless of medical knowledge. Nurses also suggested that team professionals should roster the care for hospitalized patients with mental health problems as a solution to this difficulty. However, this approach further distances nurses from the possibilities of constructing care that goes beyond the pathology related to the body^(21)^.

It is worth noting that comprehensive nursing care for hospitalized people with mental illness can be answered based on the concept of the nurse-patient relationship, understood in this study as an in-person relationship. To be developed, it requires sharing a common time and space with the other, allowing the direct experience of each other to be achieved^(10,11)^, contrary to the solution of scaling and distancing.

Nurses’ distance from care was highlighted as an unsatisfactory aspect by participants. They report that the routine to be followed, the lack of human resources, the administrative demands and the division of the nursing work process among those who care and those who manage, leave nursing technicians closer to patients, distancing nurses from care.

Such distancing can also be observed when working conditions are unfavorable for carrying out daily activities and physical facilities are inadequate, leading to constant changes in the work process^(25)^.

One possibility suggested to reduce the distance between nurses and patients is the transformation of the nursing team’s work process. It is necessary to break with the routine from care action so that new relational attitudes can be combined, implementing the in-person relationship as part of the referred care^(10,11)^.

From the transformation of nurses’ social action, their natural attitude towards the generalized phenomenon of lack of structure and bureaucratic work changes, carrying out a paradigmatic change through the stock of previously acquired knowledge. They become responsible for an active attitude and, thus, can construct new biographical aspects for the life-world of nursing care^(10,11)^.

The organization of care and work process is marked by fragmentation. This can be demonstrated in the results of this study by the moment of hospital discharge, which occurs when the subject is considered stabilized and in an ideal state of physical normality, excluding multidisciplinary care related to psychological distress, focusing only on biomedical aspects^(26)^. This form of assessment, focused only on the patient’s body, supports the lack of articulation of a care network, excluding practices and technologies related to expanded clinical knowledge necessary to guarantee comprehensive care^(21)^.

To address the effects of the fragmentation of the work and care process observed in this study, the concept of the in-person relationship is expanded to a nurse-team relationship^(10,11)^. For this relationship to happen, the nurse needs to draw on a stock of knowledge already present in his/her life-world, since teamwork is the target of nurses’ training^(10,11)^.

It is considered that, in order to organize the nurse-team relationship, aiming to distance itself from the fragmentation of practice and the biological vision focused on the disease^(27)^, nurses have a stock of prior knowledge, including training. In this study, it is recommended that such knowledge should be executed to enhance their role as major players^(10,11)^.

It is observed that the nurses in this study have their previous experiences to carry out care action^(10,11)^. Schutz defines these experiences as natural attitudes, explaining that a person orients themselves in life-world through experiences stored over time, using them to define their social reality^(10,11)^. By observing life-world, a person has the possibility of reflecting on it, analyzing it according to their way of understanding it^(10,11)^. From this perspective, nurses use their stock of knowledge built from birth to university training to read their life-world, interpret their observations, define their social reality, plan and execute the action, i.e., care^(10,11,28)^.

When referring to improvements considered essential for providing ideal care, nurses demonstrated intentions related to a broader view of patients and their health status. On the other hand, when reporting on expectations of an adequate work environment, nurses’ discourse was also permeated by a dichotomized view of a person in psychological distress, delimiting spaces of care that separate body and mind. Concerning care for a person in psychological distress from the psychosocial perspective, actions are constructed in an interdisciplinary manner, but as previously discussed and indicated in a recent study, nurses find it difficult to delimit their core actions in collective work^(29)^.

In this way, specific nursing actions are referred to as care for the body and physical health, such as checking blood pressure. In this regard, it is important that the nursing team recognizes these moments in which they carry out procedures as opportunities to establish the nurse-patient bond and relationship, understanding, from then on, that care can be carried out and sustained in the context of the in-person relationship^(10,11)^.

The nurses participating in the study also presented intentions regarding caring for a person with mental distress hospitalized due to clinical comorbidities. Among them, the expectation of having more time with patients and flexibility in routine expresses an idealization of care that aims to bring professionals closer to patients. This idealization demonstrates an opposition to the very form of action performed by them, characterized by the distance of nurses in relation to a person with mental distress and by compliance with the unit norms and routines as part of care^(24,29)^. The solutions presented by nurses for the challenges faced are linked to aspects external to their life-world, marked by the lack of leading role and empowerment of care, supporting the fragmentation already discussed^(7,30)^.

In the biomedical model in which the nurses in this study are inserted, the search for empowerment is a challenge that can be explained by the concept of natural attitude, understood as the state of consciousness in which one accepts the reality of everyday life as it presents itself^(10,11,30)^. Empowerment is a central element in nurses’ work, as it indicates professional satisfaction and can contribute to improving the care provided to people with mental health problems, naturally reaffirming their clinical role^(30)^. Thus, the life-world of nurses in this study is permeated by a daily life that is incompatible with actions related to their empowerment, which respond to the biomedical model^(10,11,30)^.

Another point highlighted refers to the questioning about their intentions regarding care for a person in psychological distress, hospitalized due to a clinical comorbidity. It was identified that there should be more preparation during and after training for the entire nursing team, demonstrating that this is an essential condition for achieving success in carrying out care action. Some changes recommended by the Brazilian National Curricular Guidelines for the Undergraduate Course in Nursing are being implemented in the curricular proposals and teaching plans, including aspects of mental health, considering them important and mandatory for training nurses^(31)^. Studies recommend strengthening nursing training in undergraduate courses, addressing mental health from the perspective of the principles of Psychiatric Reform and experiencing care devices in the territory, thus contributing to developing skills in mental health care^(32,33)^.

Nurses brought up the difficulties they faced and the feelings they felt in the nursing team, indicating their inclusion in care. Such difficulties require coping strategies, as indicated in a study conducted in Jordan with psychiatric nurses, in which they experienced moderate psychological distress and stress related to their work, requiring specific programs that promote well-being during work, reducing levels of illness^(34)^.

Furthermore, participants in this study pointed out, as a way of supporting the nursing team: the creation of spaces for sharing experiences and knowledge among professionals with experience in caring for people with mental distress; and among the team itself, for discussing issues regarding mental health, indicating the need for institutional actions related to professional appreciation as well as continuity of training^(32)^.

Finally, another aspect brought up by nurses participating in this study as necessary for providing care considered ideal is related to the multidisciplinary team. The importance of a multidisciplinary approach should be highlighted, above all, due to the aspect of comprehensiveness in health care and because it is considered a unique strategy for consolidating a new model of health care in Brazil^(35)^. The reconstruction of clinical practice in nursing work necessarily involves the concept and discussion of clinical practice, the reconstruction of in-person relationships among the subjects involved in training and production of devices that mobilize subjectivities^(29,33,35)^.

### Study limitations

The peculiarity of the institution in which it was carried out can be considered a limitation of this study, since it is a university hospital where its practices are constantly being developed with regard to nursing care.

### Contributions to health, nursing, or public policy

It is possible to point out that the results found can lead nursing professionals to reflect on their actions, enabling constructing care based on singularity, legitimizing interventions that go beyond the disease, going beyond biomedical thinking and the paradigm of disease control. It is important to highlight the development of a psychiatric nursing clinic as a possibility of overcoming fragmentation of care and favoring nurses’ work in prevention and health promotion actions, building comprehensive care.

## FINAL CONSIDERATIONS

Through Alfred Schutz’s theoretical methodological framework, it was possible to understand nurses’ experiences in caring for people with mental health problems hospitalized due to clinical comorbidities in non-psychiatric IUs. The experiences of nurses participating in this study enabled constructing a more refined view of care based on the totality of the problem addressed, making it plausible to translate the implications of the findings and their interpretations into practice and, mainly, into teaching mental health and psychiatric nursing.

Nurses’ lack of preparation to meet patients’ complexities is implicit in fragmented actions and challenges faced during hospitalization, and is related to their subordination in the biomedical model, characterized by a lack of knowledge and technical preparation. Thus, in nurses’ life-world, care action is carried out based on biographical aspects of their stock of prior knowledge. However, this action has not responded to existing demands related to people in mental distress hospitalized with clinical comorbidities, with nurses being distant from patients, dealing with bureaucratic demands of their function and having difficulty in providing care.

The intentions for carrying out ideal care demonstrated opposition to the very way in which the action is carried out, characterized by the lack of nurse leading role and empowerment and requires a paradigmatic change in nurses’ natural attitude. From this perspective, it was observed that the nurses in this study attributed the responsibility for the quality of care to factors external to their life-world, when, in fact, these aspects should be components to help them build their stock of knowledge, in order to then promote comprehensive care.

Therefore, from the perspective of ideal care, it is recommended that nursing practice be transformed based on its action, which includes aspects related to biography and knowledge stock. Moreover, new studies are suggested that seek the unique experiences lived by nursing professionals in different contexts and spaces of care.
