# Supplementary material for: Nurses’ experiences in caring for people with mental health problems hospitalized due to clinical comorbidities
Source: Rev Bras Enferm. 2024 Dec 13;77(5):e20230136. doi: 10.1590/0034-7167-2023-0136 (PMC11654220; doi:10.1590/0034-7167-2023-0136)
Supplement: Translation

# Translation: Experiências de enfermeiros no cuidado de pessoas em sofrimento psíquico hospitalizadas por comorbidades clínicas

**DOI:** 10.1590/0034-7167-2023-0136pt

**Authors:** Gabriella Paulão Previato Piton, Aldair Weber, Ana Paula Rigon Francischetti Garcia, Vanessa Pellegrino Toledo

**Affiliations:** I Universidade Estadual de Campinas. Campinas, São Paulo, Brasil

**Keywords:** Comorbidade, Assistência Integral à, Saúde, Enfermagem Psiquiátrica, Enfermeiros, Transtorno Mental

## Abstract

**Objetivos:**

compreender as experiências dos enfermeiros no cuidado da pessoa em sofrimento psíquico hospitalizada por comorbidades clínicas em Unidades de Internação não psiquiátricas.

**Métodos:**

estudo qualitativo, orientado pela fenomenologia social de Alfred Schutz. Realizaram-se 16 entrevistas fenomenológicas. O conteúdo foi analisado e discutido com base na literatura, por meio da composição de três categorias de análise.

**Resultados:**

surgiram três categorias no estudo: Desafios no cuidado enfrentados pelos enfermeiros; A ação fragmentada do cuidado; e O cuidado ideal. Desvelou-se a desarticulação da clínica, conforme descrito pelos enfermeiros, evidenciando o cuidado como uma ação afastada da integralidade da pessoa. A atuação dos enfermeiros é guiada predominantemente pelo referencial biomédico, desconsiderando a valorização da subjetividade.

**Considerações Finais:**

observou-se que o enfermeiro atribui a responsabilidade do cuidado do paciente a fatores externos ao seu mundo vida, quando, na verdade, esses aspectos deveriam ser componentes que o auxiliassem na construção de um cuidado integral.

## INTRODUÇÃO

O modelo de atenção psicossocial do Sistema Único de Saúde (SUS), constituído e implementado no contexto da Reforma Psiquiátrica, é marcado pela ampliação do conceito de processo saúde-doença, o qual considera o princípio da integralidade e do cuidado multiprofissional^(1)^. Visa buscar uma compreensão mais abrangente do ser humano, de modo que o objeto de intervenção se desloca da doença para a pessoa em sofrimento psíquico^(1)^. Nesse contexto, para garantir a integralidade do cuidado, os enfermeiros são convidados a olhar para os desafios decorrentes das demandas de saúde apresentadas pela população com transtornos mentais, vivenciando situações de cuidado para as quais os saberes têm demonstrado serem insuficientes desde a formação^(2)^.

Diante da complexidade de cuidar da pessoa em sofrimento psíquico, observa-se que essa população apresenta múltiplos desafios no cuidado, potencializados pelo risco aumentado de desenvolver comorbidades físicas decorrentes do acesso reduzido a cuidados de saúde adequados, diminuindo sua expectativa de vida^(3)^. Destaca-se que pessoas em sofrimento psíquico apresentam maior risco de mortalidade prematura devido à presença de doenças cardiovasculares, além de que conviver com um transtorno mental aumenta as chances de desenvolver uma síndrome metabólica, caracterizada por alterações no sistema cardiovascular, o que pode incluir dislipidemia, obesidade abdominal, hipertensão e hiperglicemia^(4)^. Esse risco aumentado está presente em diversas condições psiquiátricas, incluindo os transtornos depressivo maior, afetivo bipolar, esquizofrenia, ansiedade, déficit de atenção/hiperatividade e estresse pós-traumático^(4)^.

Além disso, fatores de risco para doenças cardiovasculares, tais como tabagismo, ausência de atividade física e dieta pouco saudável, são condições físicas que representam aproximadamente 70% das mortes em pacientes com esquizofrenia ou transtorno bipolar^(5)^. Assim, com as intervenções farmacológicas, observa-se importante melhora do quadro psíquico, mas o uso de medicações antipsicóticas aumenta o risco de ganho de peso, dislipidemia e diabetes mellitus, sendo esses fatores que também intensificam a possibilidade de desenvolvimento de alguma comorbidade clínica^(5)^.

A prevalência das comorbidades clínicas em pessoas em sofrimento psíquico tem sido cada vez maior em serviços de saúde, culminando em hospitalizações. Esse cenário pode apresentar repercussões no cuidado prestado, por existir baixa adesão desses pacientes ao tratamento proposto. Destacam-se, ainda, outros fatores, tais como o acesso ao serviço de saúde de forma restrita. Os problemas de saúde mental podem limitar as ações dos profissionais, no sentido de construírem práticas de cuidado integral, nas quais os princípios da inclusão e da equidade nem sempre estão garantidos para este grupo de pacientes^(6)^.

Portanto, no desafio de lidar com a complexidade de cuidar da pessoa em sofrimento psíquico e de suas comorbidades clínicas associadas, é importante conhecer a experiência das equipes de saúde no que tange ao cuidado prestado. Estudos realizados com profissionais de enfermagem brasileiros e australianos demonstram que o cuidado de pacientes psiquiátricos e não psiquiátricos hospitalizados ainda é um desafio a ser superado^(7)^. Tal desafio pode ser identificado por fatores como a rotina dos procedimentos técnicos, gerando o distanciamento do paciente devido ao estereótipo negativo da psiquiatria, e a negligência do exame físico, no caso da pessoa em sofrimento psíquico, e o exame do estado mental não é realizado nas unidades clínicas, o que pode indicar a fragmentação do cuidado tanto físico quanto mental^(7)^.

Esse cenário evidencia impactos na prática, e, desde a formação, recomenda-se a educação continuada por meio de capacitações nos distintos setores dos serviços de saúde, desde a emergência, passando pela unidade psiquiátrica até a de cuidados intensivos^(7-9)^.

Importante destacar os fatores que constituem desafios na assistência à saúde dessa população, como a fragmentação do cuidado, que envolve a complexidade da pessoa em sofrimento psíquico, o cenário epidemiológico, as implicações vivenciadas pelas equipes de enfermagem ao realizar o cuidado e existência de modelos de atenção distintos e concorrentes, um pautado no cuidado biológico e outro fundamentado na diretriz da integralidade. Nesse sentido, para avançar na busca do cuidado integral, é importante que se considere a reprodução da lógica da prática instituída, a qual é marcada pela perspectiva biológica no processo de avaliação, causando um olhar fragmentado acerca da pessoa em sofrimento psíquico acometida por uma comorbidade clínica^(1,7)^.

Este estudo é justificado pela escassez de discussão na literatura sobre os cuidados da pessoa em sofrimento psíquico hospitalizada por comorbidades clínicas. Muitos estudos são desenvolvidos com a perspectiva de obter-se dados epidemiológicos que versam sobre o seu maior risco de morte em comparação ao da população em geral^(3-6)^. É de extrema importância empreender estudos que visem conhecer as vivências dos atores envolvidos nesse cenário do cuidado, tais como os profissionais de enfermagem. Diante da baixa adesão e acesso dificultado, há impactos na prática marcados pela perspectiva biológica, como a rotina de procedimentos técnicos e o distanciamento do paciente, ocasionados pelo estereótipo da psiquiatria^(1,6-9)^.

## OBJETIVOS

Compreender as experiências dos enfermeiros no cuidado da pessoa em sofrimento psíquico hospitalizada por comorbidades clínicas em Unidades de Internação (UIs) não psiquiátricas.

## MÉTODOS

### Aspectos éticos

O estudo está em conformidade com os aspectos éticos da pesquisa com seres humanos, de acordo com a Resolução n^o^ 466/12 do Conselho Nacional de Saúde, e foi aprovado pelo Comitê de Ética em Pesquisa da Universidade Estadual de Campinas no ano de 2018. Obteve-se a anuência dos participantes ao Termo de Consentimento Livre e Esclarecido (TCLE) e ao Termo de Autorização para Gravação de Voz. Para garantir o anonimato, os participantes foram identificados pela letra “E”, de enfermeiro, seguida do número correspondente à ordem das entrevistas.

### Referencial teórico-metodológico

Na perspectiva de Alfred Schutz, a compreensão do significado da ação ocorre a partir de motivos existenciais e da ação dos sujeitos no mundo vida^(10,11)^. Ao investigá-los, encontra-se um duplo caráter motivacional fundamentado em um conjunto de “motivos para” e “motivos porque”^(10,11)^. Os “motivos porque” estão relacionados às experiências passadas, contextualizadas a partir do estoque de conhecimento do sujeito^(10,11)^. Os “motivos para” estão relacionados aos objetivos que se deseja alcançar e à intencionalidade das ações projetadas no futuro^(10,11)^. Schutz busca compreender o mundo em seu significado intersubjetivo, por meio de uma análise das relações sociais face a face, levando em consideração que as ações ocorrem de maneira consciente e possuem um significado para a pessoa^(10-12)^.

### Tipo do estudo

Trata-se de estudo qualitativo, desenvolvido a partir do referencial teórico-metodológico da fenomenologia social de Alfred Schutz^(11)^. Busca-se compreender a experiência vivenciada pelo enfermeiro no cuidado da pessoa em sofrimento psíquico hospitalizada para o tratamento de comorbidades clínicas e o significado por ele atribuído a essa ação, tomando a UI como seu mundo vida, dotado de conhecimento, subjetividade, singularidade, biografia determinada e motivações^(10-12)^. Procurando manter o rigor científico da pesquisa qualitativa, foram seguidas as recomendações do *COnsolidated criteria for REporting Qualitative research* (COREQ)^(13)^.

### Cenário de estudo

A pesquisa foi realizada em distintas UIs de um hospital público universitário, localizado no interior do estado de São Paulo, sendo esse o cenário de formação, pesquisa e extensão dos pesquisadores envolvidos neste estudo. Suas unidades ambulatoriais e de internação são formadas por profissionais diversos da equipe multiprofissional que garantem a assistência ao paciente, almejam novas formas de cuidado com base no cotidiano e ampliam a atuação da instituição com a sociedade civil. Esse hospital é referência de nível terciário para múltiplas especialidades, prestando cuidados de saúde a pacientes com distintas necessidades, os quais são acompanhados na rede pública de saúde da região metropolitana de Campinas.

### Participantes do estudo

Os participantes foram 16 enfermeiros atuantes nas UIs que vivenciaram o cuidado da pessoa em sofrimento psíquico hospitalizada para o tratamento de uma comorbidade clínica. Os critérios de inclusão foram ser enfermeiro, atuar nas UIs de adultos, cuidar de pessoas em sofrimento psíquico hospitalizadas nas UIs para o tratamento de comorbidades clínicas. Os critérios de exclusão foram ser enfermeiros que estiveram em férias ou afastamento de saúde no momento da coleta de dados.

### Coleta e organização dos dados

Para a abordagem dos participantes, foi utilizada a amostragem em rede, conhecida como bola de neve, em que um participante indica o outro^(14)^, sendo o primeiro selecionado por meio dos supervisores das UIs. Oito supervisores foram referenciados como sendo as sementes^(14)^, e esses indicaram enfermeiros que eles consideravam já terem contato com a temática do estudo. As UIs que foram cenário da coleta de dados são ortopedia, neuroclínica, gastroclínica, enfermaria geral de adultos, urgência e especialidades e moléstias infecciosas. Utilizou-se a entrevista fenomenológica para abordar os entrevistados e, baseado na intersubjetividade, foi possível ao sujeito que vivencia o fenômeno expressar o significado de sua ação^(10,12,15)^. Realizaram-se entrevistas semiestruturadas, individuais, não diretivas, que permitem a inclusão de novos questionamentos que visam explorar as experiências na totalidade. Foram gravadas com tempo médio de 40 minutos e guiadas pelas questões norteadoras: você já cuidou de pessoas em sofrimento psíquico hospitalizadas para o tratamento de comorbidades clínicas na UI de adulto? Conte-me como foi esse cuidado. Qual foi sua intenção com o cuidado realizado? Os dados foram coletados pela pesquisadora principal, que foi capacitada pela orientadora acerca da entrevista fenomenológica junto ao grupo de pesquisa. A pesquisadora não manteve vínculo direto com os enfermeiros das unidades onde os dados foram coletados. Isso possibilitou o distanciamento dos participantes e do cenário para a análise dos dados, restringindo-se apenas ao momento da coleta de dados. A coleta de dados ocorreu no local de trabalho dos enfermeiros, com data e hora pré-agendadas, de junho a setembro de 2018, sendo encerrada quando o pesquisador compreendeu que sua inquietação foi respondida e o fenômeno foi desvelado, e nenhum novo elemento emergiu das entrevistas para constituir as categorias que representam a tipificação da experiência vivida pelos enfermeiros no cuidado da pessoa em sofrimento psíquico hospitalizada com comorbidade clínica^(16,17)^. Salienta-se que todas as entrevistas foram utilizadas para a análise e discussão dos dados, não havendo perda amostral.

### Análise dos dados

Para análise dos depoimentos, foi utilizado o referencial metodológico proposto por pesquisadores da fenomenologia social^(10,12)^. Os dados foram analisados e categorizados pela pesquisadora principal e pelos demais autores que contribuíram para o estudo, sem uso de *software*. Após a transcrição das entrevistas, procedeu-se às leituras na íntegra, a fim de apreender o sentido comum da experiência do enfermeiro e caracterizar a tipificação do vivido^(10,12)^. Posteriormente, foi realizada a releitura cuidadosa das entrevistas, visando criar unidades de significado^(10,12)^. Em seguida, organizaram-se os discursos em categorias temáticas, tendo sido apreendido o significado da ação expresso pelos “motivos porque”, evidenciados pelas categorias “Desafios no cuidado enfrentados pelos enfermeiros” e “Ação fragmentada do cuidado”. Os “motivos para” foram descritos na categoria “O cuidado ideal”. Foram realizadas a análise compreensiva e a discussão dos resultados, utilizando pressupostos da fenomenologia social^(10,12)^ e da literatura científica relacionada à temática do estudo.

## RESULTADOS

### Desafios no cuidado enfrentados pelos enfermeiros

Na motivação do vivido, evidenciou-se que os participantes relataram dificuldades no cuidado da pessoa em sofrimento psíquico hospitalizada. Trata-se de uma especialidade que não é comum na UI, além de esses não terem o mesmo entendimento que outros pacientes têm sobre cuidar e se recuperar em uma internação, caracterizando-se como uma diferença na visão dos enfermeiros.

*A gente tem dificuldades, por se tratar de uma especialidade que a gente não tem todos os dias, não é. Os pacientes psiquiátricos ficam lá na enfermaria deles e, eventualmente, ficam aqui.* (E1)

*Ele não entende que tem que ficar restrito por conta da fratura que ele tem* [...] *se é uma fratura de membro inferior, é complicado, porque você vai ter que conter o paciente, senão ele se levanta e cai. Essa é a diferença desses pacientes que a gente tem.* (E8)

O discurso dos enfermeiros também demonstrou algumas dificuldades no processo de cuidar do paciente na UI. A ausência da família na internação dificulta uma relação de confiança para o cuidado e o planejamento de alta. A estrutura física inadequada também foi considerada uma dificuldade no processo de cuidar da saúde mental na UI, caracterizada pela falta de proteção, lazer e atividades fora do leito.

[…] *normalmente, estes pacientes ficam sem familiares, fica mais difícil de criar confiança, e não dá para cuidar sem que o paciente confie em você.* (E2)

*O familiar nem sempre fica presente, principalmente com o usuário de substância. Nesses casos, é mais difícil planejar a alta.* (E14)

*A estrutura física não é adequada para atender não só o paciente psiquiátrico* […] *estamos no sexto andar, sem muita proteção. Além disso, não temos nada de lazer, área externa, uma paisagem ou alguma atividade fora do leito, nada que faça de fato um cuidado com a saúde mental.* (E10)

Neste cenário, os entrevistados versam sobre o risco da descompensação do quadro psiquiátrico do paciente que se soma ao medo da equipe de enfermagem de cuidar, devido ao risco de agressão. Portanto, diante da necessidade de realizar a contenção física, os profissionais se deparam com a falta de insumos para sua efetivação, além da ausência do médico ou da prescrição médica específica, aspectos esses que os enfermeiros consideram que precisam estar presentes em protocolos de internação do paciente na UI.

[…] *se não tiver a contenção física na prescrição, dificulta muito nosso trabalho, porque, no momento da intercorrência, o médico não está* […] *sempre tem esse risco de descompensar a parte psiquiátrica, teria que ser um protocolo para a internação desse tipo de paciente.* (E7)

[…] *a equipe de enfermagem tem medo de cuidar do paciente psiquiátrico, principalmente aqueles casos que o paciente fica ameaçador.* (E8)

[…] *faltam várias coisas para cuidar do paciente psiquiátrico, material, faixas, um treinamento para contenção física.* (E5)

Vivências como a agitação psicomotora e a agressividade foram qualificadas como aspectos ruins da experiência no cuidado do paciente em sofrimento psíquico na UI, devido ao risco de contaminação, além da falta de uma equipe para responder às demandas de cuidado psicológico.

[…] *tem aquele paciente que agride funcionário, de empurrar, de querer segurar, agora mesmo tem um paciente que tirou o acesso e espirrou sangue, ele é HIV positivo* [Vírus da Imunodeficiência Humana]*, espirrou sangue no teto, espirrou na colega, foi bem ruim.* (E4)

[…] *há pacientes agressivos e agitados e, às vezes, a gente não tem uma equipe que dê para fazer esse cuidado que tem a parte psicológica.* (E3)

Por fim, os entrevistados trazem que a falta de conhecimento sobre as patologias psiquiátricas e de preparo dos enfermeiros, para cuidar da pessoa em sofrimento psíquico hospitalizada, implica questionamentos sobre o manejo, a abordagem e a condução do cuidado.

*A gente não tem um conhecimento adequado das patologias, a gente não sabe fazer esse manejo. Como abordo o paciente? Como eu cuido? Até onde posso ir?* (E12)

*Aqui é mais difícil de cuidar desses pacientes. Acho que a equipe não foi preparada para cuidar do paciente psiquiátrico, eu mesma não estive em faculdade.* (E15)

### A ação fragmentada do cuidado

Evidenciou-se que os aspectos emocionais da pessoa em sofrimento psíquico hospitalizada na UI se encontram em um plano secundário do cuidado, sendo que, durante a coleta de dados do histórico de enfermagem, o enfermeiro aponta que investiga apenas os aspectos fisiológicos, dirigindo a anamnese pelo diagnóstico da internação.

[…] *no histórico de enfermagem, tem algumas questões na abordagem emocional. Eu, por ser enfermeira de cardiologia, não dou importância para isso, eu fico no fisiológico.* (E16)

[…] *o paciente internou devido a uma TVP* [Trombose Venosa Profunda]*, então, se ele é psiquiátrico, isso acaba ficando como secundário, e os pacientes são conhecidos pelo diagnóstico da internação.* (E7)

A fragmentação da pessoa a partir das especialidades médicas retira a condição do cuidado de enfermagem referente à questão psiquiátrica durante a hospitalização em uma UI. Ainda é atribuída ao médico a solicitação de avaliação e conduta por meio de interconsulta.

*Acho que o enfermeiro da ortopedia não tem condições de cuidar do paciente psiquiátrico.* […] *mas quem tem mais experiência com essas complicações da fratura é o enfermeiro da ortopedia.* (E10)

[…] *não sei como realmente funciona a avaliação psiquiátrica. Sei que as interconsultas existem, e aí fica a critério do médico solicitar.* (E9)

Durante a internação da pessoa em sofrimento psíquico, o enfermeiro encontra dificuldade em prescrever cuidados que respondam às demandas relacionadas à saúde mental, interrogando-se como sanar a necessidade de apoio emocional. Assim, no planejamento do cuidado, os enfermeiros utilizam um rodízio entre todos os membros da equipe para que ninguém se sinta injustiçado, a fim de evitar o cansaço e o desgaste emocional.

[…] *como prescreverei para a equipe cuidar desse paciente, “dar apoio emocional” fica muito aberto. O que seria a enfermagem dar apoio emocional? Se a gente, que é enfermeiro, tem essa dificuldade de avaliar, imagina o técnico* […] *a gente não sabe exatamente o que prescrever para esse cuidado, fica confuso.* (E8)

[…] *é difícil de planejar quem vai cuidar. A gente acaba fazendo uma escala de rodízio, assim todos precisam cuidar e não fica injusto para ninguém.* (E6)

[…] *a gente visa fazer um revezamento maior dos técnicos de enfermagem, até para eles não se sentirem cansados na escala diária. A gente pensa nisso para o profissional não ter um desgaste emocional.* (E9)

Entretanto, na rotina a ser seguida na unidade, as demandas administrativas e a falta de recursos humanos prejudicam o planejamento da assistência de enfermagem. Consequentemente, os enfermeiros ficam mais distantes do cuidado, de modo que o técnico de enfermagem é o profissional que fica mais próximo.

[...] *é difícil porque já chega com uma demanda grande. A rotina passa por cima.* (E3)

[...] *ultimamente, nosso trabalho burocrático, na questão administrativa, nos levou a assumir diversas demandas de serviços, para os quais não têm mais funcionário. Então, quem está mais perto do paciente acaba dando mais esse apoio, no caso, os técnicos de enfermagem, sendo que depois eles nos passam as informações sobre o cuidado que prestaram.* (E7)

Em relação à implementação do cuidado, os entrevistados descreveram o cumprimento das prescrições médicas em horários pré-estabelecidos como o principal fator no tratamento clínico. Porém, é mencionada a possibilidade de a equipe se aproximar do paciente para realizar o cuidado em saúde mental mediante uma hospitalização prolongada.

[...] *para cumprir as prescrições médicas, o tratamento clínico, eu acredito que tenha mais peso a administração da medicação feita corretamente, prescrição correta* [...] *para a clínica, segue todos esses cuidados e horários pré-estabelecidos.* (E1)

[...] *se o paciente fica mais tempo, você consegue que a equipe converse, mas, às vezes, isso não ocorre, ele vai embora para casa sem os cuidados* [...] *fazemos, dessa forma, o cuidado da parte física e clínica, mas a parte psicológica não foi feita de forma alguma.* (E7)

### O cuidado ideal

Os enfermeiros demonstraram o desejo de terem mais tempo para conversar, escutar o paciente e sentir suas dificuldades como expectativas existentes para cuidar da pessoa em sofrimento psíquico. Somam-se a isso, juntamente, o anseio dos profissionais em ter mais preparo e formação e a necessidade do compartilhamento de vivências para a realização do cuidado ideal.

*Queria ter mais tempo de conversar, para fazer um trabalho que a gente sabe que não basta um dia, pra chegar e escutar todos os dias você tem que ficar sentindo as dificuldades.* (E1)

[...] *seria importante ter mais preparo, ter mais informações a respeito de doenças psiquiátricas. Cursos, mais informações, para estarmos mais preparados.* (E13)

*Poderia fazer uma roda, trazer um caso clínico e falar dessa parte mental* [...] *falar sobre o que é difícil, o que facilita o cuidado, pois existem pessoas que têm coisas positivas para falar, o que pode ajudar o colega.* (E3)

[...] *eu gostaria que os enfermeiros fossem capacitados em relação a essa questão do paciente psiquiátrico, capacitados em como fazer o manejo, como lidar.* (E11)

Diante disso, os entrevistados compreendem que, no momento da admissão do paciente, é necessária uma avaliação multidisciplinar, a fim de que suas necessidades em saúde sejam consideradas e, também, para que a equipe de enfermagem saiba mais sobre como cuidar.

Ademais, é identificada a necessidade de acompanhamento psicológico para a equipe de enfermagem como uma forma de cuidar de quem cuida, amparado pela peculiaridade da profissão em lidar com a morte e tragédias, fatores que podem levar ao adoecimento.

*A equipe de enfermagem, na totalidade, penso que deveria ter um acompanhamento psicológico* […] *a gente lida com morte, com várias situações assim, tragédias mesmo. Tem uma parte da equipe que já está no hospital há mais de 20 anos, então vai adoecendo.* (E4)

*Desde o momento da internação deve estar numa equipe multidisciplinar, um psiquiatra, psicólogo, terapeuta ocupacional, e que eles pudessem vir na enfermaria para avaliar o paciente nas necessidades dele* […] *seria importante essa avaliação para a equipe saber mais sobre como cuidar.* (E8)

## DISCUSSÃO

No que se refere aos desafios enfrentados pelos enfermeiros ao experienciarem o cuidado da pessoa em sofrimento psíquico hospitalizada por comorbidades clínicas, o mundo vida dos profissionais e dos pacientes possui forte influência no cuidado de enfermagem, por ter que se considerar a subjetividade de ambos^(10,11)^. O mundo vida, constituído a partir de aspectos biográficos e do estoque de conhecimento, faz-se presente na relação enfermeiro-paciente, em que ocorre a ação do cuidado como resultado de tal encontro, permeado por objetivos mútuos e previamente estabelecidos entre os principais atores^(10,11)^.

Os achados deste estudo apontam que o cuidado dos sinais e sintomas decorrentes das comorbidades clínicas é dificultado pela existência de um transtorno mental, quando há necessidade de uma internação clínica, ao trazer especificidades que não são comuns de serem encontradas nas UIs. Pode-se elencar como dificuldade a falta de entendimento sobre a necessidade de sua internação, criando-se uma barreira para oferecer cuidados clínicos. Esse cenário é resultante da fragmentação da pessoa, impactando diretamente as estratégias de cuidado e seus resultados esperados, pois o transtorno mental é visto como algo separado e não como componente do organismo biológico de um mesmo indivíduo^(7)^.

Ainda, tal fragmentação reproduz estigmas vividos e medos que são potencializados, como a agressão, atrelada ao desconhecimento de como cuidar da pessoa em sofrimento psíquico hospitalizada, dificultando a relação enfermeiro-paciente^(9,18)^. Nessa perspectiva, indiretamente, os enfermeiros entendem a importância do vínculo e trazem a presença da família durante a internação como importante estratégia para auxiliar nas distintas formas de cuidar do paciente psiquiátrico internado, devido às alterações fisiológicas^(19)^.

Outro aspecto apontado pelos enfermeiros, relacionado aos desafios no cuidado da pessoa em sofrimento psíquico hospitalizada por uma comorbidade clínica, foi o ambiente de trabalho inadequado que, segundo eles, também impacta a falta de proteção, lazer, atividades fora do leito e apoio institucional. Desse modo, é importante considerar que a ambiência da unidade de internação constitui parte do mundo vida dos enfermeiros participantes deste estudo, influenciando as relações sociais e suas atitudes que orientam como cuidar do paciente. Nesse sentido, a implementação de estratégias que melhorem a ambiência pode visar a humanização do cuidado no âmbito hospitalar^(20)^.

De acordo com a percepção dos enfermeiros envolvidos no estudo, considera-se importante que haja uma prescrição de contenção mecânica e química nos casos em que a avaliação da pessoa em sofrimento psíquico hospitalizada indique a necessidade de tal procedimento. Essa abordagem pode refletir uma visão de saúde que responde ao modelo biomédico, focado no diagnóstico psiquiátrico, deixando de lado a avaliação da condição clínica que justificaria tal prescrição e a singularidade do ser humano^(21)^.

A precipitação acerca da necessidade de protocolos de contenção, vinculados à existência do diagnóstico psiquiátrico, é sinalizada como aspecto que impacta negativamente o cuidado, configurada pela vivência dos profissionais em relação à agitação psicomotora e agressividade apresentada pela pessoa em sofrimento psíquico hospitalizada. A violência e a agitação, quando consideradas no mundo vida, constituídas pelo estoque de conhecimentos e os aspectos biográficos dos enfermeiros, podem justificar o distanciamento da subjetividade e da singularidade de cada pessoa, no qual o cuidado de enfermagem é legitimado pelo saber científico, impondo um estado de normalidade ideal e saudável acerca de como a pessoa deve ser e se comportar^(10,11,22)^.

A partir de Schutz, pode-se dizer que não existiu a aquisição de conhecimentos relacionados ao cuidado da pessoa em sofrimento psíquico hospitalizada como parte do mundo vida dos enfermeiros estudados em sua construção do estoque de conhecimento, caracterizado pela falta de preparo técnico-científico durante o período de formação, bem como na perspectiva da educação continuada. Essa lacuna na formação se encontra implícita na dificuldade de o enfermeiro realizar uma avaliação integrada acerca do estado de saúde da pessoa em sofrimento psíquico hospitalizada por uma comorbidade clínica^(7,10,11)^.

Nesse sentido, estudos brasileiros apontaram as dificuldades no cuidado da pessoa em sofrimento psíquico hospitalizada, pois, mesmo em serviços especializados no atendimento dessa população, foi identificado um déficit em relação ao conhecimento técnico-científico específico para capacitar futuros profissionais para o manejo em saúde mental. Foram, ainda, observadas barreiras relacionadas aos processos de trabalho, tais como o esgotamento das equipes, o sucateamento dos serviços e o distanciamento dos profissionais com o paciente^(21,23)^.

Também foi possível constatar que os enfermeiros reproduziam o olhar reducionista e fragmentado da patologia pautado em um processo biológico, separando corpo e mente ao realizarem a admissão do paciente na UI: baseavam-se apenas nas etapas do exame físico, excluindo o exame do estado mental, distanciando-se da avaliação do paciente sobre os aspectos que vão além do corpo. Tal achado corrobora estudo brasileiro que evidenciou o distanciamento do enfermeiro em relação ao paciente psiquiátrico hospitalizado, deixando de realizar o exame físico e de sistematizar o cuidado de saúde mental do paciente clínico através do exame de estado mental^(7)^.

Ainda, ao conhecer a percepção dos enfermeiros sobre os sintomas psiquiátricos do paciente clínico, esses relatam baixa carga horária na formação, identificando a necessidade de qualificar o cuidado por meio de capacitações, conhecendo unidades de internações psiquiátricas^(24)^. Nesse sentido, evidencia-se que a atenção aos sintomas psiquiátricos da pessoa em sofrimento psíquico hospitalizada ocorre em um plano secundário ao cuidado clínico, caracterizando a fragmentação do cuidado, identificada pela ausência de aspectos relacionados ao estoque de conhecimento dos enfermeiros sobre a enfermagem de saúde mental e psiquiátrica e sua prática profissional, predominando conceitos de senso comum e do cotidiano do mundo vida social^(7,10,11,24)^.

A vivência descrita pelos enfermeiros sinaliza o mundo vida da enfermagem com o cuidado da pessoa em sofrimento psíquico hospitalizada. Esse mundo é dividido entre os profissionais da equipe, a fim de que o enfermeiro permaneça em sua situação biográfica já existente e constituída, ainda que seja identificada a necessidade de se motivar em busca de elementos para o estoque de conhecimento^(10,11)^. As emoções e sentimentos trazidos pelos entrevistados em seus relatos sobre a experiência com esse cuidado, tais como o medo de ser agredido e o desconforto em relação ao paciente psiquiátrico, podem configurar a relação face a face, caracterizada como o encontro com o mundo vida de outro sujeito, orientando-se reciprocamente com maior possibilidade de compreensão mútua^(9-11)^. Para o cuidado de enfermagem da pessoa em sofrimento psíquico hospitalizada, a relação face a face de Schutz se mostra como possibilidade de minorar os desconfortos vividos pelos enfermeiros na relação enfermeiro-paciente. Permite compreender que as sensações permeiam tal relação a partir do contato com o mundo vida e seus diversos aspectos constituintes até o momento deste encontro^(10,11)^.

Nesse paradigma, os discursos dos enfermeiros participantes do estudo também apontaram a dificuldade de planejar e prescrever um cuidado de enfermagem que inclua a saúde mental do paciente, independentemente do saber médico. Os enfermeiros também sugeriram a realização de um rodízio dos profissionais da equipe no cuidado da pessoa em sofrimento psíquico hospitalizada como solução para essa dificuldade, porém tal recurso aprofunda o distanciamento dos enfermeiros das possibilidades de construir um cuidado para além da patologia relacionada ao corpo^(21)^.

Destaca-se que o cuidado integral de enfermagem da pessoa em sofrimento psíquico hospitalizada pode ser respondido com base na concepção da relação enfermeiro-paciente, compreendida neste estudo, como relação face a face. Para ser desenvolvida, precisa da partilha com o outro de um tempo e espaço em comum, permitindo o alcance da experiência direta uma da outra^(10,11)^, contrárias à solução do escalonamento e distanciamento.

O afastamento do enfermeiro do cuidado foi apontado como um aspecto insatisfatório pelos participantes. Relatam que a rotina a ser seguida, a falta de recursos humanos, as demandas administrativas e a divisão do processo de trabalho da enfermagem, entre quem cuida e quem gerencia, deixam o técnico de enfermagem mais próximo do paciente, distanciando o enfermeiro do cuidado.

Tal distanciamento pode ser observado também quando as condições de trabalho são desfavoráveis para a realização de atividades cotidianas e as instalações físicas se mostram inadequadas, levando a constantes alterações no processo de trabalho^(25)^.

Uma possibilidade sugerida para diminuir a distância do enfermeiro do paciente é a própria transformação do processo de trabalho da equipe de enfermagem. Faz-se necessário um rompimento com a rotina a partir da ação do cuidado, para que se possa conjugar novas atitudes relacionais, implementando a relação face a face como parte do referido cuidado^(10,11)^.

A partir da transformação da ação social do enfermeiro, muda sua atitude natural em relação ao fenômeno generalizado da falta de estrutura e do trabalho burocrático, realizando uma mudança paradigmática por meio do estoque de conhecimento previamente adquirido. Torna-se responsável por uma atitude ativa e, assim, pode construir novos aspectos biográficos para o mundo vida do cuidado de enfermagem^(10,11)^.

A organização do cuidado e do processo de trabalho é marcada pela fragmentação. Pode ser demonstrada no resultado deste estudo pelo momento da alta hospitalar, que ocorre quando o sujeito é considerado estabilizado e sob um estado ideal de normalidade física, excluindo o cuidado multiprofissional referente ao sofrimento psíquico, focando somente nos aspectos biomédicos^(26)^. Essa forma de avaliação, voltada apenas ao corpo do paciente, corrobora a falta de articulação de uma rede de cuidados, excluindo práticas e tecnologias relativas a um conhecimento clínico ampliado e necessário para a garantia da assistência integral^(21)^.

Para enfrentar os efeitos da fragmentação do processo de trabalho e do cuidado observados neste estudo, amplia-se o conceito da relação face a face para uma relação enfermeiro-equipe^(10,11)^. Para que essa relação aconteça, o enfermeiro precisa lançar mão de um estoque de conhecimento já presente no seu mundo vida, uma vez que o trabalho em equipe é alvo da formação dos enfermeiros^(10,11)^.

Considera-se que, para organizar a relação enfermeiro-equipe, visando distanciar-se da fragmentação da prática e da visão biológica focada na doença^(27)^, o enfermeiro tem estoque de conhecimento prévio, incluindo a formação. Neste estudo, recomenda-se que tal conhecimento deva ser executado para potencializar o seu papel de protagonista^(10,11)^.

Observa-se que os enfermeiros deste estudo dispõem de suas experiências prévias para realizar a ação do cuidado^(10,11)^. Schutz define essas experiências como atitudes naturais, explicando que uma pessoa se orienta no mundo vida por meio das experiências armazenadas ao longo do tempo, utilizando-as para definir sua realidade social^(10,11)^. Ao observar o mundo vida, a pessoa tem a possibilidade de refletir sobre ele, analisando-o de acordo com sua forma de compreendê-lo^(10,11)^. Sob essa perspectiva, o enfermeiro utiliza seu estoque de conhecimento construído desde o nascimento à formação na universidade para fazer uma leitura do seu mundo vida, interpretar suas observações, definir sua realidade social, planejar e executar a ação, qual seja, o cuidado^(10,11,28)^.

Ao referirem melhorias consideradas essenciais para a realização de um cuidado ideal, os enfermeiros evidenciaram intencionalidades relacionadas a um olhar mais ampliado acerca do paciente e de seu estado de saúde. Por outro lado, ao relatarem sobre as expectativas de um ambiente de trabalho adequado, o discurso dos enfermeiros também foi permeado por um olhar dicotomizado sobre a pessoa em sofrimento psíquico, delimitando espaços de cuidado que separam corpo e mente. No que tange ao cuidado da pessoa em sofrimento psíquico na perspectiva psicossocial, as ações são construídas de forma interdisciplinar, mas, como discutido anteriormente e indicado em estudo recente, os enfermeiros encontram dificuldades em delimitar as suas ações de núcleo no trabalho coletivo^(29)^.

Dessa maneira, as ações específicas da enfermagem são referidas como cuidado com o corpo e a saúde física, por exemplo, ao verificar pressão arterial. Neste sentido, é importante que a equipe de enfermagem reconheça esses momentos em que desenvolve procedimentos como oportunidades para o estabelecimento do vínculo e da relação enfermeiro-paciente, compreendendo, a partir de então, que o cuidado pode ser realizado e sustentado no contexto da relação face a face^(10,11)^.

Os enfermeiros participantes do estudo também apresentaram intencionalidades em relação ao cuidado com a pessoa em sofrimento psíquico hospitalizada por comorbidades clínicas. Entre elas, a expectativa de ter mais tempo com o paciente e de flexibilização na rotina expressa uma idealização de cuidado que visa à aproximação dos profissionais com o paciente. Essa idealização demonstra uma oposição à própria forma da ação realizada por eles, caracterizada pelo distanciamento dos enfermeiros em relação à pessoa em sofrimento psíquico e pelo cumprimento das normas e rotinas da unidade como parte do cuidado^(24,29)^. As soluções apresentadas pelos enfermeiros para os desafios enfrentados são ligadas a aspectos externos ao seu mundo vida, marcadas pela falta de protagonismo e empoderamento do cuidado, corroborando a fragmentação já discutida^(7,30)^.

No modelo biomédico em que o enfermeiro deste estudo está inserido, a busca pelo empoderamento é um desafio que pode ser explicado pelo conceito de atitude natural, compreendido como o estado de consciência em que se aceita a realidade do cotidiano da vida conforme ele se apresenta^(10,11,30)^. O empoderamento é elemento central para o trabalho do enfermeiro, ao indicar a satisfação do profissional, e pode contribuir para a melhoria do cuidado prestado às pessoas em sofrimento psíquico, reafirmando de forma natural o seu papel clínico^(30)^. Neste sentido, o mundo vida dos enfermeiros deste estudo é permeado por um cotidiano incompatível com ações relativas ao seu empoderamento, as quais respondem ao modelo biomédico^(10,11,30)^.

Outro ponto destacado se refere ao questionamento sobre suas intencionalidades em relação ao cuidado da pessoa em sofrimento psíquico hospitalizada por uma comorbidade clínica. Identificou-se que deve haver mais preparo durante e após a formação para toda a equipe de enfermagem, demonstrando que essa é uma condição imprescindível para a obtenção do sucesso na realização da ação do cuidado. Algumas mudanças preconizadas pelas Diretrizes Curriculares Nacionais do Curso de Graduação em Enfermagem estão sendo implementadas nas propostas curriculares e nos planos de ensino, incluindo aspectos de saúde mental, considerando-os importantes e obrigatórios para a formação dos enfermeiros^(31)^. Estudos recomendam o fortalecimento da formação dos enfermeiros na graduação, abordando a saúde mental na perspectiva dos princípios da Reforma Psiquiátrica e vivenciando os dispositivos de cuidado em território, contribuindo, dessa forma, para desenvolver habilidades na assistência em saúde mental^(32,33)^.

Os enfermeiros trouxeram as dificuldades enfrentadas e os sentimentos despertados na equipe de enfermagem, indicando sua inclusão nos cuidados. Tais dificuldades requerem estratégias de enfrentamento, assim como indicado em estudo realizado na Jordânia com enfermeiros psiquiátricos, em que esses experimentaram sofrimento psíquico moderado e estresse relacionado à ocupação, necessitando de programas específicos que promovam o bem-estar durante o trabalho, reduzindo os níveis de adoecimento^(34)^.

Além disso, os participantes deste estudo apontaram, como forma de apoio à equipe de enfermagem: a criação de espaços para o compartilhamento de vivências e saberes entre profissionais com experiência no cuidado de pessoas em sofrimento psíquico; e entre a própria equipe, para a discussão de questões acerca de saúde mental, indicando a necessidade de ações institucionais relacionadas à valorização do profissional, assim como da continuidade da formação^(32)^.

Por fim, outro aspecto trazido pelos enfermeiros participantes deste estudo como necessário para a realização de um cuidado considerado ideal é referente à equipe multiprofissional. A importância da abordagem multidisciplinar deve ser ressaltada, sobretudo, pelo aspecto da integralidade no cuidado em saúde e por ser considerada uma estratégia ímpar para a consolidação de um novo modelo de atenção à saúde no Brasil^(35)^. A reconstrução da prática clínica no trabalho do enfermeiro passa necessariamente pelo conceito e discussão de clínica, pela reconstrução das relações face a face entre os sujeitos envolvidos na formação e pela produção de dispositivos mobilizadores de subjetividades^(29,33,35)^.

### Limitações do estudo

Pode-se considerar como limitação deste estudo a peculiaridade da instituição em que foi realizada, já que se trata de um hospital universitário onde suas práticas estão em constante construção no que se refere ao cuidado de enfermagem.

### Contribuições para a área da saúde, enfermagem ou políticas públicas

É possível apontar que os resultados encontrados podem levar os profissionais de enfermagem a reflexões sobre suas ações, possibilitando a construção do cuidado com base na singularidade, legitimando intervenções que vão além da doença, ultrapassando o pensamento biomédico e o paradigma do controle das patologias. É importante destacar o desenvolvimento da clínica da enfermagem psiquiátrica como uma possibilidade de superar a fragmentação do cuidado e de favorecer o trabalho do enfermeiro em ações de prevenção e promoção da saúde, construindo um cuidado integral.

## CONSIDERAÇÕES FINAIS

Por meio do referencial teórico metodológico de Alfred Schutz, foi possível compreender as experiências dos enfermeiros no cuidado da pessoa em sofrimento psíquico hospitalizada por comorbidades clínicas em UIs não psiquiátricas. As vivências dos enfermeiros participantes deste estudo viabilizaram a construção de um olhar mais refinado do cuidado baseado na totalidade da problemática abordada, tornando plausível a translação das implicações dos achados e suas interpretações para a prática e, principalmente, para o ensino de enfermagem de saúde mental e psiquiátrica.

O despreparo do enfermeiro em responder às complexidades do paciente está implícito nas ações fragmentadas e nos desafios enfrentados na hospitalização, tendo relação com sua subordinação no modelo biomédico, caracterizado pela falta de conhecimento e preparo técnico. Assim, no mundo vida do enfermeiro, a ação do cuidado é realizada com base nos aspectos biográficos do seu estoque de conhecimento prévio. Porém, essa ação não tem respondido às demandas existentes relacionadas às pessoas em sofrimento psíquico hospitalizadas com comorbidades clínicas, com o enfermeiro estando distante do paciente, ocupando-se de demandas burocráticas da função e com dificuldade de realizar o cuidado.

As intencionalidades para a realização do cuidado ideal demonstraram oposição à própria forma como a ação é realizada, caracterizada pela falta de protagonismo, empoderamento do enfermeiro e requer uma mudança paradigmática na atitude natural dos enfermeiros. Sob essa perspectiva, observou-se que os enfermeiros deste estudo atribuíam a responsabilidade da qualidade do cuidado aos fatores externos ao seu mundo vida, quando, na verdade, esses aspectos deveriam ser componentes a auxiliá-los na construção do seu estoque de conhecimentos para, então, promover um cuidado integral.

Portanto, na perspectiva do cuidado ideal, recomenda-se a transformação da prática do enfermeiro fundamentada em sua ação, o que inclui aspectos relacionados à biografia e ao estoque de conhecimento. Além disso, sugerem-se novos estudos que busquem as experiências singulares vividas pelos profissionais de enfermagem em distintos contextos e espaços de cuidado.
